# Association of Opioid Dependence with the Clinical Performance of Intrathecal Ropivacaine During Spinal Anesthesia: A Prospective Cohort Study

**DOI:** 10.5812/aapm-173621

**Published:** 2026-06-30

**Authors:** Majid Razavi, Bentolhoda Ebrahimi, Ebrahim Golmakani, Monavar Afzal Aghaee, Mehryar Taghavi Gilani

**Affiliations:** 1 Department of Anesthesiology, Imam Reza Hospital, Faculty of Medicine, Mashhad University of Medical Sciences Mashhad Iran; 2 Social Determinants of Health Research Center, Faculty of Medicine, Mashhad University of Medical Sciences Mashhad Iran

**Keywords:** Opioid Dependence, Spinal Anesthesia, Ropivacaine, Recovery Room

## Abstract

**Background:**

Opioid dependence may be associated with altered pain processing and may influence the efficacy of local anesthetics during spinal anesthesia.

**Objectives:**

This study evaluated the association of opioid dependence with sensory and motor block characteristics and early postoperative analgesic outcomes following intrathecal ropivacaine anesthesia.

**Methods:**

In this prospective cohort study, 60 patients undergoing surgery under spinal anesthesia were enrolled, including 30 opioid-dependent patients and 30 non-dependent controls. Opioid dependence was characterized by a history of regular use of heroin-containing opioid preparations, with recent exposure confirmed by blood-based screening. All participants received intrathecal 0.5% ropivacaine (8 - 10 mg) with fentanyl 25 μg. Sensory block onset and duration, time to maximum sensory block, motor block characteristics, recovery-room pain scores, rescue analgesia, hemodynamic parameters, adverse events, and patient satisfaction were assessed.

**Results:**

Ultimately, 28 patients in each group were analyzed. No statistically significant differences in baseline demographic characteristics were observed between the groups. Opioid-dependent patients had shorter sensory and motor block durations (P = 0.003 and P = 0.001, respectively), longer sensory block onset time (P = 0.001), and longer time to maximum sensory block (P = 0.002). Motor block onset and time to maximum motor block did not differ significantly between the groups. Recovery-room pain scores were higher at several assessment time points in the opioid-dependent group, and 11/28 (39.3%) patients in this group required morphine, compared with 4/28 (14.3%) controls (P = 0.035). No significant differences were observed in overall patient satisfaction or adverse events. Hemodynamic measurements did not differ significantly at most time points; however, systolic blood pressure was higher at 25 minutes and heart rate was lower at 12 minutes after spinal anesthesia in the opioid-dependent group (both P = 0.039).

**Conclusions:**

Opioid dependence was associated with shorter sensory and motor block durations, a longer sensory block onset time, and greater analgesic requirements in the early recovery room after spinal anesthesia with intrathecal ropivacaine. These findings suggest that opioid-dependent patients may benefit from individualized perioperative anesthetic and analgesic strategies to optimize pain management and potentially improve clinical outcomes.

## 1. Background

Spinal anesthesia is often preferred over general anesthesia in appropriate patients because of its favorable perioperative profile, particularly in older patients with obstructive or restrictive pulmonary diseases. The level, quality, onset, and duration of sensory block, as well as the degree of motor block, are influenced by multiple factors, including age, height, weight, local anesthetic characteristics, patient positioning, and the surgical procedure (1, 2).

In opioid-dependent individuals, physiological and neuroadaptive changes involving opioid receptor signaling, density, and sensitivity may alter pain perception and responses to analgesic interventions. Chronic opioid exposure has been associated with altered analgesic responses, including opioid tolerance and resistance (3-5). Along with individuals with substance dependence, patients with chronic pain may develop opioid tolerance and dependence. These changes may influence perioperative pain assessment and analgesic requirements (3, 6, 7).

Previous studies have reported differences in the characteristics of spinal anesthesia among chronic opioid users or opioid-dependent patients, although findings have varied according to the local anesthetic, opioid exposure pattern, surgical procedure, and outcome definition (8-11). These observations raise the possibility that chronic opioid exposure may be associated with differences in the clinical characteristics of spinal anesthesia and with increased resistance to its effects.

Spinal anesthesia may be particularly relevant in this population because some patients with chronic opioid exposure may also have medical consequences of substance use. Therefore, careful perioperative assessment and appropriate postoperative analgesic planning are important in this group.

Ropivacaine has pharmacological properties similar to those of bupivacaine but generally produces less intense motor blockade and has a favorable cardiovascular safety profile. Its clinical characteristics in spinal anesthesia have been described in nonobstetric and obstetric populations (12-14).

## 2. Objectives

Although previous studies have evaluated spinal anesthesia in opioid-dependent patients, data on intrathecal ropivacaine remain relatively limited. Therefore, this study aimed to evaluate the association between opioid dependence and sensory and motor block characteristics and early postoperative analgesic outcomes after spinal anesthesia with intrathecal ropivacaine.

## 3. Methods

The study protocol was approved by the Research Council and Ethics Committee of Mashhad University of Medical Sciences (ethics code: IR.MUMS.REC.1402.087). Official ethics approval information is available through the institutional ethics review system [https://ethics.research.ac.ir/EthicsProposalView.php?id=339387]. This prospective cohort study was conducted at an educational hospital in Mashhad, Iran, between 2023 and 2024. A total of 60 patients were included in the data collection, with 30 participants in each group. Written informed consent was obtained from all participants before enrollment.

Eligible participants were aged 18 - 70 years, had an American Society of Anesthesiologists physical status of I - II, and were scheduled for surgery under spinal anesthesia. Opioid dependence was defined on the basis of a self-reported history of regular use of heroin-containing opioid preparations and ongoing use. The routes of use recorded in the study were inhalational and oral, and recent exposure was confirmed through blood-based screening using morphine and methadone screening kits approved by the Iranian Ministry of Health. Participants reported continued opioid use until the day before surgery. Exclusion criteria were hypersensitivity to local anesthetics; contraindications to spinal anesthesia, including coagulopathy or infection at the puncture site; uncontrolled systemic disease; cardiovascular instability; and failed spinal anesthesia.

Eligible patients were approached consecutively during the study period. Opioid-dependent patients were enrolled on the basis of a documented history of regular use of heroin-containing opioid preparations and positive blood screening. Nondependent controls were selected from patients without a history of opioid use who were scheduled for similar surgical procedures under spinal anesthesia. Although the groups were assembled to obtain comparable age and sex distributions, formal prospective matching (individual or frequency matching) was not performed. After the initial enrollment of 30 participants in each group, 2 patients from each group were excluded post hoc to improve balance in age and sex, yielding a final analytic sample of 28 patients per group. The flow of participants through the study is shown in Figure 1.

**Figure 1.**
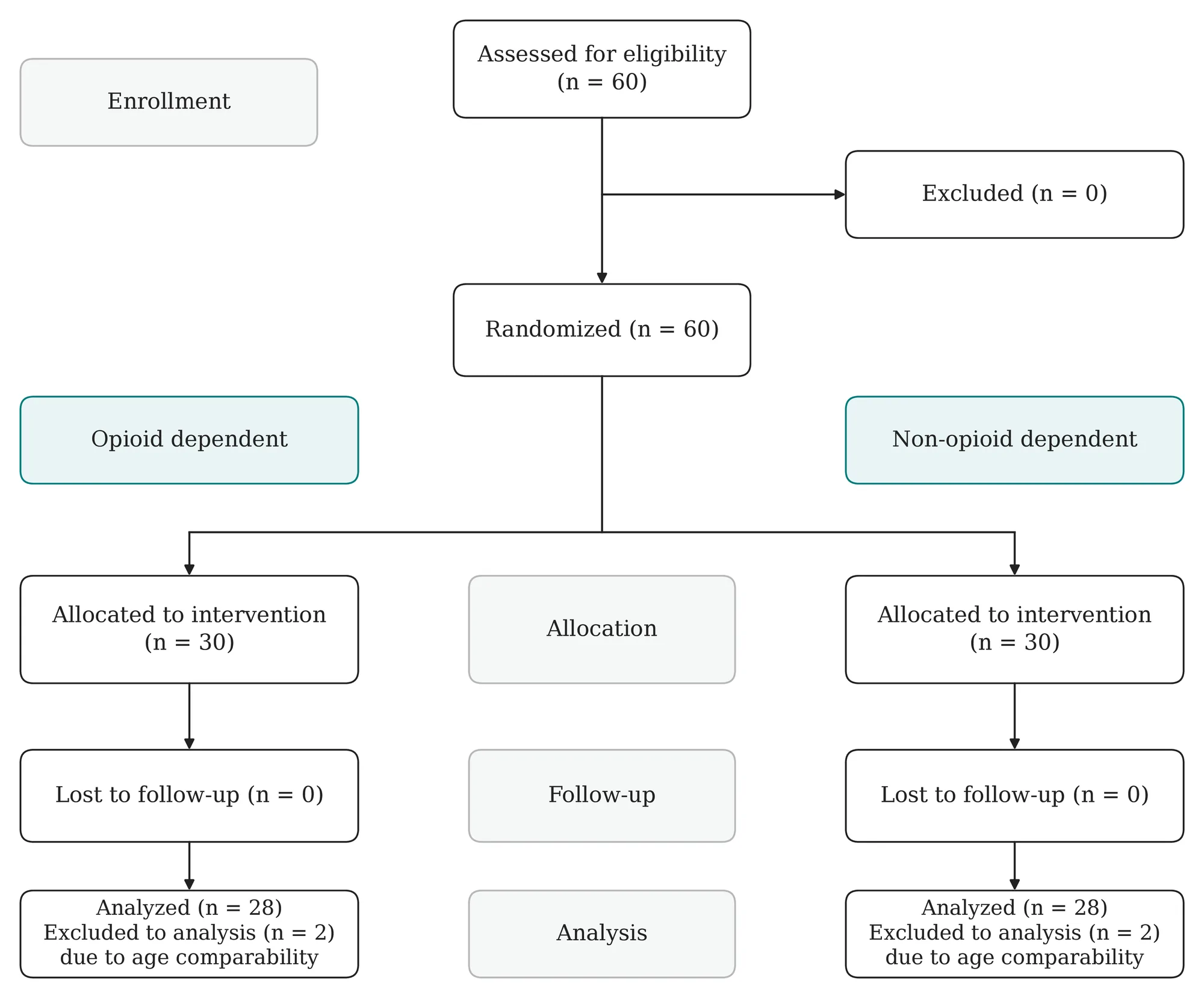
Participant flow through enrollment, group allocation, follow-up, and analysis in the prospective cohort study. Thirty patients were included in each group; 2 patients per group were excluded from analysis, leaving 28 per group. No patients were lost to follow-up.

Intravenous access was established, and participants received 250 - 500 mL of saline. Standard monitoring included noninvasive blood pressure, electrocardiography, and heart rate monitoring. Spinal anesthesia was performed in the sitting position at the L3 - L4 or L4 - L5 intervertebral space using a 25-gauge Quincke spinal needle. All patients received 8 - 10 mg of 0.5% ropivacaine combined with 25 μg of fentanyl intrathecally. The ropivacaine dose was selected according to the anticipated duration of surgery and body height. Following injection, patients were placed supine. Blood pressure and heart rate were recorded every 3 minutes during the first 15 minutes and subsequently every 10 minutes. Hypotension was defined as a ≥ 30% reduction from baseline or systolic blood pressure < 90 mmHg and was treated with intravenous ephedrine.

Sensory spread was assessed using bilateral pinprick testing along the midline dermatomes. The sensory level was defined as the most cephalad dermatome at which a clear reduction in pinprick sensation was detected compared with baseline sensation. The highest sensory level achieved during the observation period was recorded as the maximum sensory spread. Motor block was assessed using the modified Bromage scale, ranging from 0 (no motor block; full movement of the lower limbs) to 3 (complete motor block; inability to move the ankle, knee, or hip). Motor block onset time, corresponding to the time to maximum motor block, was defined as the interval from completion of the intrathecal injection to attainment of a Bromage score of 3. Motor block duration was defined as the interval from attainment of a Bromage score of 3 until regression to a Bromage score of 0.

The primary outcome was sensory block duration, defined as the interval from attainment of the T10 sensory level to regression of the sensory block to the T12 dermatome. The sensory level was reassessed every 15 minutes until regression to T12. Secondary outcomes included sensory block onset time, defined as the interval from completion of the intrathecal injection to the first detectable sensory loss on pinprick testing. Sensory block was assessed by pinprick testing every 30 seconds until the block reached T10 and then T6 to assess cephalad spread.

Additionally, time to maximum sensory block, motor block characteristics, recovery-room pain scores, rescue analgesia, hemodynamic parameters, adverse events, and patient satisfaction were assessed. Recovery-room pain was assessed using the numeric rating scale, ranging from 0 to 10, at recovery-room admission (baseline) and at 3, 6, 9, 12, 15, 25, 35, 45, and 55 minutes after admission. Intravenous morphine 5 mg was administered when the numeric rating scale score was > 4. Pain scores and the time of morphine administration were recorded for each group. The primary comparison of rescue analgesia focused on the proportion of patients who required at least 1 dose of morphine rather than on total opioid consumption. Patient satisfaction was assessed using a 3-point Likert scale (fair, good, and bad) before transfer to the ward.

Two anesthesiologists who were aware of group allocation performed all spinal anesthesia procedures. Data collection and postoperative assessments were performed by an anesthesiology resident who was blinded to group allocation. The patients were unaware of the study hypothesis and outcome assessments.

### 3.1. Statistical Analysis

The sample size was calculated from the findings of a previous study (15), using sensory block duration as the primary parameter. Assuming a two-sided α level of 0.05 and statistical power of 80%, the required sample size was estimated to be 25 participants per group. Because the exact mean and standard deviation (SD) values used in the original calculation were not recoverable from the available documentation, the calculation was based on the reported difference in sensory block duration and the stated power assumptions. To allow for potential attrition and incomplete data, the sample size was increased to 30 participants per group, resulting in a total planned sample size of 60.

Statistical analyses were performed using SPSS version 20. Continuous variables were summarized as mean ± SD or median and interquartile range, as appropriate, and categorical variables as frequencies and percentages. Normality was assessed using the Kolmogorov–Smirnov test. Between-group comparisons used the independent-samples t-test or Mann–Whitney U test for continuous variables and the chi-square or Fisher exact test for categorical variables. The primary analysis focused on sensory block duration. No imputation of missing data was performed. Analyses were conducted using available observations for each outcome. Complete data were available for the primary outcome (sensory block duration) in all 56 patients. For recovery-room pain scores, missing observations at individual time points ranged from 0 to 2 patients per group. No failed spinal blocks occurred after enrollment; patients in whom an adequate sensory level could not be achieved were excluded before group allocation according to the predefined exclusion criteria, as shown in Figure 1.

Pain intensity was repeatedly assessed at multiple time points in the same participant; these observations were considered correlated within individuals and analyzed using a linear mixed-effects model, with group (opioid-dependent vs. opioid non-dependent), time, and the group-by-time interaction included as fixed effects and participant as a random effect. The group-by-time interaction was assessed using repeated-measures ANOVA to evaluate the pattern of pain scores over time. Two-sided P < 0.05 was considered statistically significant.

## 4. Results

A total of 60 patients were initially enrolled, with 30 patients in each group. To improve comparability between the groups with respect to age and sex, 2 patients from each group were excluded from the final analysis, leaving 28 patients per group (56 patients in total). No patients were lost to follow-up after the intervention. The primary analysis was performed using the complete-case population for each outcome. Participant flow is illustrated in Figure 1.

Baseline demographic characteristics are shown in Table 1. No statistically significant differences were observed between the groups in age, sex, weight, height, or ASA status. Surgical procedures included hernia repair in 43 patients (76.8%), transurethral resection of the prostate in 11 patients (19.6%), and knee prosthesis surgery in 2 patients (3.6%). The distribution of surgical procedures was comparable between the groups (P = 0.54). Intrathecal ropivacaine was administered at a mean dose of 9.6 ± 0.41 mg in the opioid-dependent group and 9.5 ± 0.52 mg in the non-dependent group. Most patients in both groups received 10 mg (opioid-dependent: 22/28, 78.6%; non-dependent: 20/28, 71.4%), whereas the remaining patients received 8 mg.

**Table 1.** Comparison of Demographic Characteristics Between Opioid-Dependent and Non-dependent Groups ^a^

Parameters	Opioid-dependent (n = 28)	Non-dependent (n = 28)	P-Value
**Age (y)**	41.71 ± 10.38	40.42 ± 14.73	0.11
**Sex F/M**	1/27	2/26	0.56
**Weight (kg)**	62.28 ± 12.34	69.50 ± 9.37	0.06
**Height (cm)**	167.82 ± 5.53	169.30 ± 6.37	0.61
**ASA physical status**			0.29
I	23	25	
II	5	3	

^a^ Values are presented as mean ± SD or Number. Abbreviations: ASA, American Society of Anesthesiologists; F/M, female/male.

Opioid-dependent patients had shorter sensory and motor block durations (P = 0.003 and P = 0.001, respectively) and a longer sensory block onset time (P = 0.001). Time to maximum sensory block was also longer in the opioid-dependent group (P = 0.002). Neither motor block onset time (2.69 ± 1.23 vs 2.69 ± 1.11 min, respectively; P = 0.151) nor time to maximum motor block (2.77 ± 1.75 vs 2.68 ± 1.60 min, respectively; P = 0.076) differed significantly between the opioid-dependent and non-dependent groups (Table 2). The final sensory block level showed significantly greater cephalad spread in opioid-dependent patients than in non-dependent patients (P = 0.01). Sensory block levels reaching T6 were observed more frequently in the opioid-dependent group.

**Table 2.** Comparison of Sensory and Motor Block Characteristics Between Opioid-Dependent and Non-dependent Groups ^a^

Parameters (min)	Opioid-dependent	Non-dependent	P-Value
**Sensory block duration**	106.54 ± 29.61	131.24 ± 26.21	0.003
**Sensory block onset time**	2.60 ± 1.13	2.42 ± 0.90	0.001
**Time to reach maximum sensory block**	2.74 ± 1.15	2.20 ± 1.29	0.002
**Motor block duration**	112.13 ± 32.52	139.91 ± 37.72	0.001
**Motor block onset time**	2.69 ± 1.23	2.69 ± 1.11	0.151
**Time to reach maximum motor block**	2.77 ± 1.75	2.68 ± 1.60	0.076

^a^ Values are presented as mean ± SD.

Pain scores recorded in the recovery room were generally higher in the opioid-dependent group than in the non-dependent group at several assessment time points (Table 3). No significant between-group differences were observed at admission (baseline) or 3, 6, or 9 minutes after admission. Pain scores were higher in the opioid-dependent group at 12, 15, 25, 35, 45, and 55 minutes after admission. Pain intensity changed significantly over time, and the pattern differed between groups, as indicated by the group-by-time interaction. Post hoc comparisons showed significantly higher pain scores in the opioid-dependent group starting 12 minutes after recovery-room admission. The first dose of morphine was administered 12 minutes after admission in the opioid-dependent group and 25 minutes after admission in the non-dependent group.

**Table 3.** Comparison of Recovery-Room Pain Scores (NRS score, 0-10) Between Opioid-Dependent and Non-dependent Groups ^a^

**Time After Recovery-Room Admission (min)**	Opioid-dependent	Non-dependent	P-Value
**Admission (baseline)**	1.24 ± 0.10	0.91 ± 0.64	0.321
**3**	1.50 ± 1.50	0.75 ± 1.10	0.510
**6**	1.46 ± 1.42	0.89 ± 1.10	0.138
**9**	1.96 ± 1.50	1.60 ± 1.16	0.432
**12**	3.03 ± 1.64	1.75 ± 1.26	0.021
**15**	3.42 ± 1.42	2.57 ± 0.95	0.036
**25**	3.96 ± 1.20	3.14 ± 1.35	0.025
**35**	3.89 ± 1.39	3.17 ± 1.30	0.042
**45**	4.28 ± 1.38	3.21 ± 1.47	0.006
**55**	4.60 ± 1.39	3.64 ± 1.85	0.033

^a^ Values are presented as mean ± SD. Pointwise comparisons are exploratory. Abbreviation: NRS, numeric rating scale.

Table 4 summarizes postoperative rescue analgesia requirements and patient satisfaction. Rescue analgesia with intravenous morphine was required in 11 of 28 patients (39.3%) in the opioid-dependent group, compared with 4 of 28 patients (14.3%) in the control group (P = 0.035). Most participants in both groups reported good satisfaction. Bad satisfaction was reported by 5 (17.9%) opioid-dependent patients and 7 (25.0%) non-dependent patients; good satisfaction, by 19 (67.9%) patients in each group; and fair satisfaction, by 4 (14.3%) and 2 (7.1%) patients, respectively. The overall distribution of satisfaction did not differ significantly between the groups (P = 0.067).

**Table 4.** Comparison of Rescue Analgesia Requirement and Patient Satisfaction Between Opioid-Dependent and Non-dependent Groups ^a^

Parameters	Opioid-dependent (n = 28)	Non-dependent (n = 28)	P-Value
**Rescue analgesia**			0.035
Yes	11 (39.3)	4 (14.3)	
No	17 (60.7)	24 (85.7)	
**Patient satisfaction**			0.067
Bad	5 (17.9)	7 (25.0)	
Good	19 (67.9)	19 (67.9)	
Fair	4 (14.3)	2 (7.1)	

^a^ Values are presented as No. (%).

Hemodynamic parameters were comparable between the groups at most assessment time points (Figure 2). No statistically significant between-group differences in systolic or diastolic blood pressure were observed at most time points. Systolic blood pressure was significantly higher in the opioid-dependent group 25 minutes after spinal anesthesia (P = 0.039). Heart rate was significantly lower in the opioid-dependent group 12 minutes after spinal anesthesia (P = 0.039), with no statistically significant between-group differences at the other assessment time points. Intravenous ephedrine was administered to 1 patient in each group.

**Figure 2.**
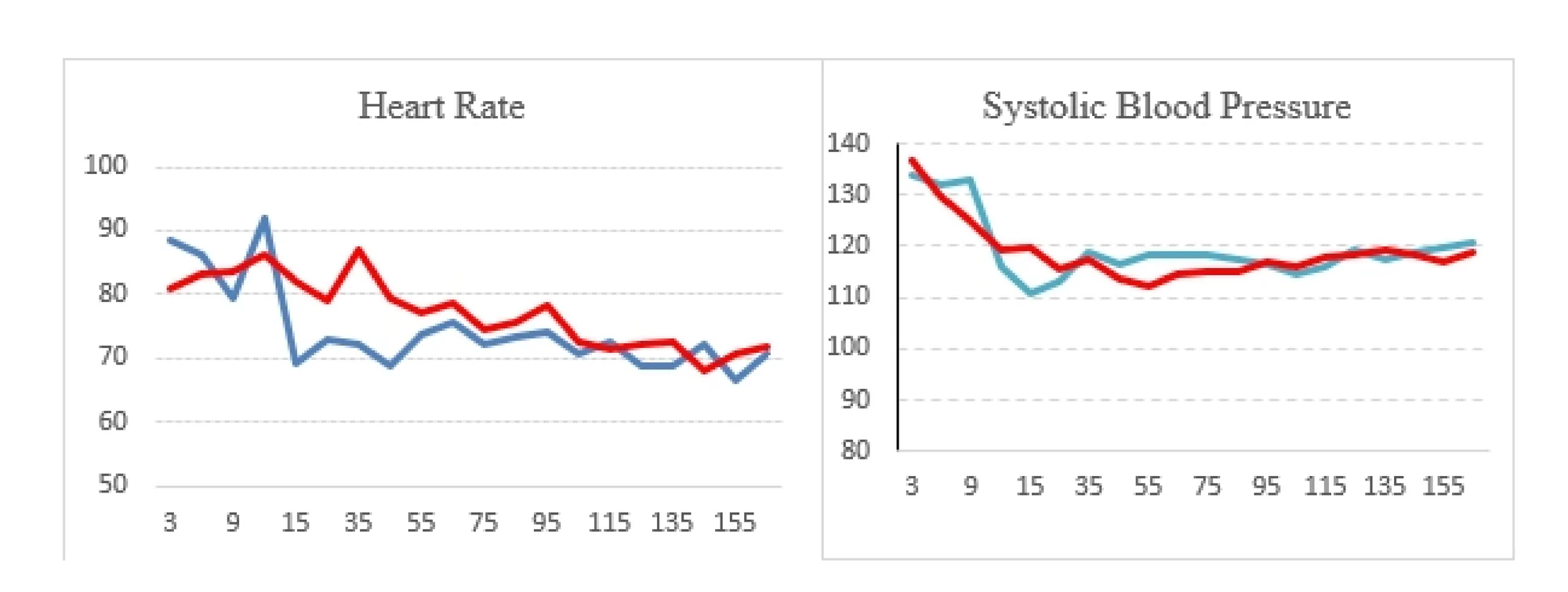
Changes in systolic blood pressure and heart rate during the perioperative period. Systolic blood pressure was significantly higher in the opioid-dependent group at 25 minutes (P = 0.039), and heart rate was significantly lower at 12 minutes (P = 0.039). No other between-group differences were statistically significant. The opioid-dependent group is shown in blue and the non-dependent group in red.

## 5. Discussion

The present study evaluated the characteristics of spinal anesthesia with intrathecal ropivacaine in opioid-dependent and non-dependent patients undergoing surgery. Compared with non-dependent patients, opioid-dependent patients had significantly shorter sensory and motor block durations, longer sensory block onset times and longer times to maximum sensory block, higher recovery-room pain scores, and a greater proportion of patients requiring rescue analgesia. No statistically significant between-group differences were observed in motor block onset time, time to maximum motor block, recorded adverse events, or overall patient satisfaction. Hemodynamic parameters did not differ significantly at most time points, except for higher systolic blood pressure at 25 minutes and lower heart rate at 12 minutes after spinal anesthesia in the opioid-dependent group. These findings suggest an association between opioid dependence and differences in sensory block characteristics and early postoperative pain management following spinal anesthesia with ropivacaine.

An important finding of the present study was the higher pain intensity observed during recovery-room assessments in opioid-dependent patients. Although no statistically significant between-group differences were observed at baseline or at 3, 6, and 9 minutes, pain scores were higher in the opioid-dependent group at several subsequent assessment time points, from 12 to 55 minutes after recovery-room admission. Although previous studies have not specifically evaluated recovery-room pain following spinal anesthesia with intrathecal ropivacaine in opioid-dependent patients, altered pain processing and increased analgesic requirements have been described in individuals with chronic opioid exposure. Proposed mechanisms include opioid tolerance, opioid-induced hyperalgesia, and neuroadaptive changes involving both peripheral and central pain-processing pathways (16-18). These mechanisms may contribute to the greater early postoperative analgesic needs observed in opioid-dependent patients in the present study.

Several mechanisms may contribute to the differences in pain intensity and rescue analgesic requirements observed in opioid-dependent patients after surgery. Chronic opioid exposure is associated with neuroadaptive changes in opioid receptor signaling, particularly involving μ-opioid receptor pathways. Alterations in endogenous opioid systems and central pain-processing mechanisms may also influence responses to analgesic interventions (3, 4, 19). In the present study, the greater proportion of patients requiring rescue morphine and the higher recovery-room pain scores in the opioid-dependent group may be consistent with these previously described neuroadaptive changes. However, because opioid receptor function, opioid-induced hyperalgesia, and other neurobiological mechanisms were not directly assessed in this study, these mechanisms should be regarded as possible explanations rather than established causes of the observed differences.

Another important finding of the present study was the longer sensory block onset time and longer time to maximum sensory block in opioid-dependent patients. These findings indicate an association between opioid dependence and differences in the early development of sensory blockade following intrathecal administration of ropivacaine. However, the underlying mechanisms remain unclear. Alterations in neuronal excitability, opioid-related neuroadaptation, and potential interactions between opioid signaling pathways and the pharmacological effects of local anesthetics have been proposed as possible explanations for differences in the response to spinal anesthesia in patients with chronic opioid exposure (19, 20). However, these mechanisms were not directly evaluated in the current study.

Previous investigations have reported differences in spinal block characteristics among opioid-dependent or chronic opioid-using patients. Vosoughian et al. reported a shorter duration of sensory blockade following intrathecal lidocaine administration in opioid-dependent patients (8). Dabbagh et al. similarly reported shorter sensory and motor block durations after intrathecal bupivacaine in chronic opium users (9), whereas Mansourian et al. found a shorter duration of spinal anesthesia in opioid-dependent patients receiving different local anesthetic agents (10). Karbasy et al. also reported lower sensory block levels following intrathecal 0.5% bupivacaine in chronic opium users undergoing lower abdominal and lower extremity surgery (11). Although these studies generally suggest differences in spinal anesthesia characteristics among patients with chronic opioid exposure, their findings cannot be directly equated with the present results because of differences in local anesthetic agents, study populations, opioid exposure patterns, surgical procedures, and outcome definitions. In particular, the present study evaluated ropivacaine and assessed sensory block duration, onset time, and time to maximum sensory block. Therefore, the current findings should be interpreted as an observed association rather than as evidence that opioid dependence directly reduces the effectiveness of spinal anesthesia.

Unlike a previous spinal-anesthesia study that reported no significant difference in sensory block onset between opioid-dependent and non-dependent patients (16), the present study found longer sensory block onset times and longer times to maximum sensory block in opioid-dependent patients. A separate study of erector spinae plane block before general anesthesia also found no significant between-group difference in block onset (21); however, that study involved a different regional anesthetic technique. Several factors may account for differences among studies, including opioid exposure characteristics and patterns, local anesthetic selection and dosing, anesthetic techniques, surgical procedures, and definitions used to assess block onset and time to maximum block. Most previous investigations of spinal anesthesia have focused on bupivacaine or lidocaine, whereas the present study used ropivacaine (8-11). In addition, the ropivacaine dose in the current study was determined according to the anticipated duration of surgery rather than patient body weight. These methodological differences should be considered when comparing the present findings with those of previous studies.

The present study also found a greater need for rescue analgesia among opioid-dependent patients. Intravenous morphine was required by 39.3% of opioid-dependent patients, compared with 14.3% of controls. This finding is consistent with the higher recovery-room pain scores observed in the opioid-dependent group and may reflect altered pain processing or opioid tolerance associated with chronic opioid exposure. Therefore, the present findings should be interpreted as a difference in the proportion of patients requiring rescue analgesia rather than as evidence of a difference in total postoperative opioid requirements. Previous reports have also described increased perioperative analgesic requirements among patients with chronic opioid exposure (7); however, differences in patient populations, opioid exposure, anesthetic techniques, and postoperative analgesic protocols limit direct comparison with the present study.

Despite the differences observed in recovery-room pain and sensory block characteristics, overall patient satisfaction did not differ significantly between the groups. Satisfaction is a multidimensional outcome that may be influenced by pain, expectations, communication with the clinical team, the perioperative experience, and other psychosocial factors. The absence of a significant difference in satisfaction despite higher pain scores in the opioid-dependent group suggests that satisfaction may not be determined by early postoperative pain alone.

### 5.1. Limitations

This study has several limitations that should be considered when interpreting the findings. First, the observational cohort design does not permit causal inference, and residual confounding cannot be excluded. Although the groups were assembled to improve comparability, formal prospective matching was not performed, and detailed information on several potentially relevant confounders was limited. Second, opioid exposure was characterized by clinical history and blood-based confirmation of recent exposure, but quantitative information regarding daily opioid dose, cumulative duration of use, exact timing of the last use, maintenance treatment, and polysubstance exposure was unavailable. Third, the dose of intrathecal ropivacaine was selected according to the anticipated duration of surgery and body height, and the study included different types of surgery. Thus, variation in ropivacaine dose and surgical characteristics may have contributed to the observed differences. Finally, the relatively small sample size and single-center setting may limit the precision and generalizability of the findings. Larger prospective studies using standardized diagnostic criteria, quantitative characterization of opioid exposure, standardized surgical and anesthetic protocols, and appropriate longitudinal and multivariable analyses are warranted to confirm these observations.

### 5.2. Conclusions

In this prospective cohort study, opioid-dependent patients undergoing spinal anesthesia with intrathecal ropivacaine had shorter sensory and motor block durations, longer sensory block onset time, and longer time to achieve maximum sensory block than non-dependent patients. They also had higher pain scores during recovery-room assessment and were more likely to require rescue morphine. These findings suggest an association between opioid dependence and differences in the early characteristics of sensory blockade and rescue analgesic requirements after spinal anesthesia with ropivacaine. Consequently, anesthesiologists should consider these altered physiological and pharmacological responses when planning perioperative pain management strategies for patients with chronic opioid dependence. Future multicenter studies with larger sample sizes are warranted to further investigate the association between opioid dependence and spinal anesthesia characteristics and to determine optimal anesthetic strategies for this growing patient population.

## Data Availability

The data supporting the findings of this study are not publicly available because they contain participant-level information and are subject to ethical and privacy considerations. De-identified data may be made available from the corresponding author upon reasonable request, subject to approval by the relevant institutional authorities and ethics requirements.
